# AmpliSeq Screening of Genes Encoding the C-Type Lectin Receptors and Their Signaling Components Reveals a Common Variant in *MASP1* Associated with Pulmonary Tuberculosis in an Indian Population

**DOI:** 10.3389/fimmu.2018.00242

**Published:** 2018-02-20

**Authors:** Tilman E. Klassert, Surabhi Goyal, Magdalena Stock, Dominik Driesch, Abid Hussain, Luis Carlos Berrocal-Almanza, Rajashekar Myakala, Gaddam Sumanlatha, Vijayalakshmi Valluri, Niyaz Ahmed, Ralf R. Schumann, Carlos Flores, Hortense Slevogt

**Affiliations:** ^1^ZIK Septomics, Jena University Hospital, Jena, Germany; ^2^Institute of Microbiology and Hygiene, Charité Universitätsmedizin Berlin, Berlin, Germany; ^3^BioControl Jena GmbH, Jena, Germany; ^4^Department of Biotechnology and Bioinformatics, University of Hyderabad, Hyderabad, India; ^5^Mahavir Hospital & Research Center, Hyderabad, India; ^6^CIBER de Enfermedades Respiratorias, Instituto de Salud Carlos III, Madrid, Spain; ^7^Research Unit, Hospital Universitario N.S. de Candelaria, Universidad de La Laguna, Santa Cruz de Tenerife, Spain; ^8^Genomics Division, Instituto Tecnológico y de Energías Renovables (ITER), Santa Cruz de Tenerife, Spain

**Keywords:** C-type lectin receptor, MASP1, pulmonary tuberculosis, complement, AmpliSeq

## Abstract

Tuberculosis (TB) is a multifactorial disease governed by bacterial, host and environmental factors. On the host side, growing evidence shows the crucial role that genetic variants play in the susceptibility to *Mycobacterium tuberculosis* (Mtb) infection. Such polymorphisms have been described in genes encoding for different cytokines and pattern recognition receptors (PRR), including numerous Toll-like receptors (TLRs). In recent years, several members of the C-type lectin receptors (CTLRs) have been identified as key PRRs in TB pathogenesis. Nevertheless, studies to date have only addressed particular genetic polymorphisms in these receptors or their related pathways in relation with TB. In the present study, we screened the main CTLR gene clusters as well as CTLR pathway-related genes for genetic variation associated with pulmonary tuberculosis (PTB). This case-control study comprised 144 newly diagnosed pulmonary TB patients and 181 healthy controls recruited at the Bhagwan Mahavir Medical Research Center (BMMRC), Hyderabad, India. A two-stage study was employed in which an explorative AmpliSeq-based screening was followed by a validation phase using iPLEX MassARRAY. Our results revealed one SNP (rs3774275) in *MASP1* significantly associated with PTB in our population (joint analysis *p* = 0.0028). Furthermore, serum levels of MASP1 were significantly elevated in TB patients when compared to healthy controls. Moreover, in the present study we could observe an impact of increased MASP1 levels on the lectin pathway complement activity *in vitro*. In conclusion, our results demonstrate a significant association of *MASP1* polymorphism rs3774275 and MASP1 serum levels with the development of pulmonary TB. The present work contributes to our understanding of host-Mtb interaction and reinforces the critical significance of mannose-binding lectin and the lectin-complement pathway in Mtb pathogenesis. Moreover, it proposes a *MASP1* polymorphism as a potential genetic marker for TB resistance.

## 1 Introduction

Tuberculosis (TB) remains a major global health problem affecting millions of people each year and ranking as the first leading cause of death from an infectious disease worldwide (1). *Mycobacterium tuberculosis* (Mtb), a highly successful intracellular pathogen, is transmitted typically through aerosols into the respiratory system, thereby developing an infection. It has been well established that both innate and adaptive immune responses are required for host control of tuberculosis infection (2). In TB pathogenesis, the host cellular immune response determines whether an infection is arrested as latent or persistent infection or progresses to the next stages, i.e., the active TB infection (3). Efficient cell-mediated immunity hinders tuberculosis infection by permanently arresting the infection at latent or persistent stage, but if the initial infection in the lung is not controlled or if the immune system becomes weakened, Mtb can cause active pulmonary and to a lesser extend extra-pulmonary tuberculosis (4).

Several pattern recognition receptors (PRRs) expressed on various immune cells play a major role in the recognition of Mtb and transduce signals either directly *via* receptor ligation or through various adaptor molecules to initiate an appropriate immune response (5). The PRR family of Toll-like receptors (TLRs) has been well described for their contribution to the Mtb-associated immune responses (6–9). In addition, the family of C-type lectin receptors (CTLRs) has been recently discovered to also recognize Mtb, leading to a considerable modulation of Mtb-induced immune responses and have secured a prominent and ongoing spot in TB research. Potent Mtb associated molecular patterns, including trehalose-6,6-dimycolate (TDM) and mannose-capped lipoarabinomannan (ManLAM), are recognized by CTLRs such as Mincle, MCL, and Dectin-2 (10–13). Moreover, Dectin-1 has been shown to be important for generating reactive oxygen species and other proinflammatory responses (14–16), while the mannose-binding lectin (MBL) interacts with Mtb directly to activate the lectin pathway of the complement system (17). Therefore, CTLRs binding to Mtb are associated with the induction or the modulation of several important signaling pathways such as the Syk-CARD9-Bcl10-MALT1 pathway, phagosome maturation, and complement activation (18–22).

Susceptibility to Mtb has a definite genetic component and host-genetic variation is thought not only to determine infection outcome, but also the risk of disease progression (3). Therefore, variants of genes involved in innate host-defense mechanisms have been associated with host susceptibility to TB (23). Various genome-wide association studies and candidate-gene studies demonstrate that several single nucleotide polymorphisms (SNPs) in certain genes are associated with TB susceptibility (24–26). In particular, SNPs in TLRs and their pathway adaptors have been widely associated with TB (27, 28). Additionally, particular SNPs in genes of the CTLR family have been investigated in case–control studies and found to be associated with TB susceptibility as reviewed in Goyal et al. (11). These include variants in the genes encoding for MBL and MASP2 (29, 30), which play a major role in the activation of the lectin complement pathway (Figure S1 in Supplementary Material). However, comprehensive studies addressing susceptibility to Mtb in association with genetic variants in the entire set of CTLR genes and their related pathways have not been performed so far. Here, we aimed to identify specific SNPs in the genes of CTLRs or/and in the genes of the related pathway adaptors that may have an impact on TB susceptibility and/or disease severity in a well-phenotyped Indian population (31, 32) from Hyderabad, where the TB prevalence is very high (33). An AmpliSeq-based approach was used as innovative technique in a two-stage process to screen for relevant polymorphisms in 33 genes. In this study, we identified an intronic SNP in the MBL-associated serine protease (*MASP1*) gene, an important component of the lectin pathway of the complement, associated with pulmonary tuberculosis (PTB) infection in our population.

Identification of genetic variations among genes of the CTLR pathways that influence the susceptibility to TB may lead to a better understanding of the pathogenesis and the development of novel strategies for the prevention and treatment of this significant infectious disease.

## 2 Material and Methods

### 2.1 Subject and Samples

We carried out a case–control study to determine whether common variants in genes involved in CTLR-dependent responses might be associated with the development of PTB in an Indian population. For that purpose, 144 PTB case patients and 181 unrelated healthy controls were recruited at the Mahavir Hospital and Research Center in Hyderabad (India) between July 2011 and November 2013. Criteria for inclusion of cases were: (i) admission in the Hyderabad Directly Observed Treatment, Short-course (DOTS) program at Mahavir Hospital, and (ii) new diagnosis of pulmonary sputum smear positive TB disease. The diagnostic criterion for PTB was defined as the presence of one of the following: at least 2 initial sputum smear examinations positive for Acid-Fast Bacilli (AFB) or sputum examination positive for AFB and radiographic abnormalities consistent with active PTB (34). Criteria for inclusion as healthy control were: (i) absence of apparent acute or chronic pulmonary diseases or diseases of other origin, (ii) clinically in good health at the time of enrollment, and (iii) a negative history of TB disease. All subjects were from the same geographical origin, and residing in Hyderabad.

### 2.2 Ethics Statement

All study participants gave written informed consent in accordance with the Declaration of Helsinki. The study was approved by the institutional ethics committee for bio-medical research at the Bhagwan Mahavir Medical Research Center, Hyderabad, India (date: March 11, 2011).

### 2.3 Study Design

This study was divided in two phases: a genomic stage with an AmpliSeq-based discovery approach followed by a validation through iPLEX MassARRAY genotyping of candidate SNPs. In Phase 1 (discovery), we used 40 samples from each cases and controls group for Next Generation Sequencing (NGS)-profiling of selected regions (detailed in the AmpliSeq Library Preparation section below). For this initial phase, the 40 case samples were chosen based on the severity of the disease (determined by the chest X-rays and sputum microscopy). The population characteristics are provided in Table 1. After NGS, association analysis was performed at allele and genotype level. Candidate SNPs were then subjected to a second phase (validation), comprising the genotyping of all remaining cohort samples for the candidate SNPs obtained from the explorative approach. The genotyping was performed using the MassARRAY iPLEX Platform (Agena Bioscience).

**Table 1:** Summary of case–control study characteristics.

Parameter	Cases (*n* = 144)	Controls (*n* = 181)	*p*-Value
Age (years)	27 ± 11	31 ± 10	0.0008
Gender (M/F)	71/73	103/78	0.1808
BMI (kg/m^2^)	16 ± 2.6	24 ± 4.7	<2.2e−16
Smoking (yes/no)	32/112	29/151	0.1980

*Statistical analysis was performed using T-test (for age and BMI) and exact Fisher-test (for gender and smoking)*.

### 2.4 DNA Extraction

Peripheral blood samples were collected from 144 PTB patients and 181 unrelated healthy controls. DNA was isolated from blood samples using QIAamp DNA Blood Mini Kit (Qiagen; Hilden-Germany) following manufacturer’s instructions. DNA samples were stored at −20°C until further usage.

### 2.5 Discovery Phase

#### 2.5.1 AmpliSeq Library Preparation

The targets for this study included the C-type lectin receptor genes encoded in two gene clusters of chromosome 12: the Dectin-1 cluster (221 kb, comprising *MICL, CLEC2, CLEC9A, CLEC12B, CLEC1, Dectin-1*, and *LOX1*), and the Dectin-2 cluster (812 kb, comprising *BDCA2, DCIR, Dectin-2, MCL*, and *MINCLE*). Entire genes (including introns and exons) as well as several intergenic regions (including 1 kb of the 5′ flanking regions of all genes) of both clusters were selected for sequencing. In addition, an extensive literature search was performed to ensure the inclusion of SNPs in other CTLRs or adaptors in their signaling pathways that have been already associated with PTB and/or fungal infections as well as lung infections (35–48) as targets in our AmpliSeq panel (see Table S1 in Supplementary Material for final targets list). This selection was supplemented with Tag-SNPs, SNPs that were informative of common gene variation, of the other important CTLR receptors/adaptors. Tag-SNPs lists were extracted from the UCSC Genome Browser (https://genome.ucsc.edu/), using the Affymetrix Genome-Wide Human SNP Array 6.0 (Assembly GRCh37/hg19) as reference. All targeted regions were encoded in a bed file for megaplex primer pair design using the AmpliSeq Designer version 3.0.1 (Thermo Fisher Scientific, USA). The design resulted in 83% effective coverage of the targeted regions. The final AmpliSeq design (Table S1 in Supplementary Material) comprised 1,470 amplicons, with expected amplicon sizes between 125 and 275 bp, divided in two pools of 739 and 731 amplicons.

DNA-AmpliSeq libraries were prepared using the Ion AmpliSeq™ Library Kit 2.0 (Thermo Fisher Scientific, USA), following manufacturers’ instructions. In brief, 10 ng of DNA (for each pool) from 80 samples (40 cases/40 controls) were used as input for the HiFi-amplification with the designed primer mix. Resulting PCR products were subjected to partial primer digestion using FuPa reagent and subsequently ligated to barcoded Ion adapters (Ion Xpress™ Barcode Adapters Kit; Thermo Fisher Scientific, USA). The Ion Library Equalizer Kit was used to normalize library concentration to 100 pM, and AmpliSeq libraries were pooled for sequencing.

#### 2.5.2 Sequencing

Library template pools were clonally amplified on Ion Sphere particles using the Ion PI™ Template OT2 200 Kit v2 on the instrument Ion OneTouch™ 2 System (Thermo Fisher Scientific, USA). The sequencing chips were prepared using the Ion PI Sequencing 200 Kit v2 (Thermo Fisher Scientific, USA), and sequenced on an Ion Proton Sequencer (Thermo Fisher Scientific, USA). In total, 80 samples were multiplexed on 2 chips for sequencing. The raw sequence data in bam format have been stored in the Sequence Read Archive (SRA) at National Center for Biotechnology Information (NCBI), and can be accessed at NCBI homepage (https://www.ncbi.nlm.nih.gov/; accession number: SRP123407).

#### 2.5.3 SNP Identification

The quality of the raw data in fastq format was checked using FastQC (http://www.bioinformatics.babraham.ac.uk/projects/fastqc) and, thereafter, adapter sequences and low-quality regions (Phred Q score < 20) were trimmed using cutadapt (49). The trimmed reads for each sample were mapped onto the hg19 reference genome with Bowtie2 (50).

To identify SNPs, the Variant Caller plugin of the Partek Genomics Suite 6.6 (Partek Inc., St Louis, MO, USA) was used. SNPs were then kept for further analyses according to a minimal sequencing depth (>20 reads/sample), Minor Allele Frequency (MAF > 0.02) and the existence of Hardy-Weinberg Equilibrium (HWE) in control samples. All known biallelic SNPs passing these filters were then subjected to association analyses.

#### 2.5.4 Association Study

In order to control population stratification in the discovery phase, we used the LASER (Locating Ancestry from SEquence Reads) v. 2.01 software (51). All entries corresponding to the superpopulation code SAS (South Asian) were obtained from the 1000 Genomes Project (ftp://ftp.1000genomes.ebi.ac.uk/vol1/ftp/release/20130502/). These included datasets from the following populations: Gujarati (GIH), Punjabi (PJL), Bengali (BEB), Sri Lankan Tamil (STU), and Telugu (ITU). All known SNPs annotated by the 1000 Genomes Project were retrieved as vcf files and filtered for the amplicon regions covered in our AmpliSeq approach using VCFtools (52) and for a MAF > 0.05 using the GenomeAnalysisToolKit (GATK v. 3.2) (53, 54). The filtered SNP list was then pruned using PLINK (55) in order to exclude less informative SNPs in linkage disequilibrium (LD). A total of 161 resulting SNPs from the reference populations were then used as input for LASER (51) to define the PCA space and derive the background coordinates for ancestry adjustment.

Using PLINK (55), we used logistic regression models for association analysis of genotypes under the assumption of an additive inheritance model, using the first coordinate to adjust for population stratification. Success of stratification correction was empirically assessed *via* quantile-quantile plots and statistical inflation estimates (lambda) using the gap package (56, 57) for R (58); R Development Core Team, 2013; http://www.R-project.org/. Chi-square tests were also applied to test for allele frequency differences.

### 2.6 Validation Phase

#### 2.6.1 iPLEX MassARRAY Genotyping

All SNPs with a statistical significance for association in the discovery (*p* < 0.2), and a nominal significance (*p* < 0.05) in the allele frequency distribution between groups were kept for follow-up studies in the validation phase. In this stage, 245 additional samples (141 controls and 104 TB patients) from the cohort were analyzed by iPLEX MassARRAY at Agena Bioscience GmbH (Hamburg). Statistical analysis of all samples was performed using logistic regressions with PLINK (55).

### 2.7 Meta-Analysis of Association Results

METAL (59) was used to combine the *per* SNP results from association studies in the discovery and validation phases. For this, the joint analysis used the *p*-values across the two phases taking sample size and direction of effect into account.

### 2.8 Measurement of MASP1, MASP3, and MAp44 Levels in Serum

The serum concentrations of MASP1, MASP3, and MAp44 in 106 healthy controls and 99 TB patients were measured using commercially available ELISA kits following the manufacturer’s instructions (Human MASP1 ELISA Kit, Cloud-Clone Corp., Human MASP3 ELISA Kit, Hycult biotech Inc., Human MAp44 ELISA Kit, Hycult biotech Inc.). Diluted serum samples were incubated in the coated plates for the recommended time period and the amount of protein sandwiched was detected by a conjugated antibody and subsequent measurement of absorbance at 450 nm.

### 2.9 Measurement of Lectin Pathway Complement Activity

Serum from healthy donor blood samples was obtained by short centrifugation at 3,000 *g* at 4°C for 10 min. The serum MASP1 and MBL levels were measured by ELISA (Human MASP-1 ELISA Kit, Cloud-Clone Corp., Hycult Biotech Human MBL ELISA kit). To investigate the effect of increased MASP1 levels on complement function, recombinant human MASP1 (Creative BioMart MASP1-137H) was added at different concentrations (+13% rhMASP1, +26% rhMASP1, or +52% rhMASP1) to the donor serum, and complement activity after MBL pathway activation was measured using a commercially available ELISA kit (Complement system MBL pathway WIESLAB^®^) following manufacturer’s instructions. Briefly, six diluted serum samples were measured in duplicate along with blank, positive and negative controls, and incubated at 37°C for 1 h. After washing, the formation of terminal complement complex C5b-9 was detected using conjugated antibody and absorbance was measured at 405 nm on a microplate reader (TECAN SpectraFluor Plus).

## 3 Results

### 3.1 Discovery Study (Phase I)

In the discovery phase of this study, we screened the Dectin-1 and Dectin-2 gene clusters, as well as other CTLR-relevant genomic regions for potential variants that might be associated with pulmonary tuberculosis in an Indian population. Our AmpliSeq design covered 83% of the targeted regions, and yielded over six hundred known SNPs that passed the filters for sequencing depth, MAF and HWE (Table S2 in Supplementary Material). Since a heterogeneous ancestral background can be presumed for our study population (Figure S2A in Supplementary Material), we corrected for potential stratification effects using the first coordinate derived by the LASER v2.1 software (51).

No inflation of association results was evident based on quantile-quantile plots and lambda results (*lambda* = 1.004; see Figure S2B in Supplementary Material). After association analysis, we selected 18 common variants as candidate SNPs for follow up studies in the next phase (Table S3 in Supplementary Material). These included 2 exonic SNPs in *CD207* (chr. 2), 1 SNP in *MASP1* (chr. 3), 1 SNP in *SFTPA1* (chr. 10), and 14 SNPs in CTLRs of the Dectin-clusters in chromosome 12, including an intronic variant in *CLEC7A* (*Dectin1*), a missense variant in *CLEC1B*, and several variants in *CLEC12A* (*MICL*) and *CLEC12B*. All these variants showed differences at nominal significance level in their allele frequency distribution between cases and controls, and yielded top *p*-values (cutoff *p* < 0.2) when addressing their genotype distribution after ancestry adjustment using logistic regression models (Table S3 in Supplementary Material).

### 3.2 Validation (Phase II): Rs3774275 in MASP1 Is Significantly Associated with TB

Phase II of the study consisted in a MassARRAY-based genotyping of a total of 245 independent samples (141 controls and 104 TB patients) addressing the aforementioned candidate variants. Primer design failed for four of the 18 selected variants, which were excluded from the validation by the MassARRAY. SNP rs374147676 was monomorphic in the samples from this phase and was removed from further analyses. Among the remaining 13 common variant candidates, only one SNP (rs3774275; *MASP1*) was nominally significant in phase II (*p* = 0.0340, Table 2), showing the same direction of effects as in phase I.

**Table 2:** List of common variants subjected to validation through MassARRAY, showing the results of the association analysis after phase II, as well as the joint analysis performed integrating data from phases I and II.

SNP_ID	Location				Type of mutation	Alleles		Association (*p*-values)		Effect
	Gene	Chr	Position (GRCh38)	Gene location		Test allele	Other	Phase II MassARRAY	Meta-analysis	
rs741326	*CD207*	2	70831704	Exonic	Missense	**A**	G	0.6727	0.1292	Risk
rs2080390	*CD207*	2	70831095	Exonic	Synonym.	**T**	C	0.7578	0.1726	Risk
rs3774275	*MASP1*	3	187247480	Intronic	Intron var.	**G**	A	0.0340*	0.0028**	Protective
rs1914663	*SFTPA1*	10	79612197	Intronic	Intron var.	**T**	C	0.9470	0.2902	Protective
rs76427726	*CLEC12A*	12	9950609	Intronic	Intron var.	**C**	T	0.0995	0.5581	Risk
rs35333643	*CLEC12A*	12	9957832	Intronic	Intron var.	**G**	A	0.2096	0.9281	Risk
rs148864420	*CLEC12A*	12	9959987	Intronic	Intron var.	**A**	C	0.2497	0.8767	Protective
rs648985	*CLEC12A*	12	9963978	Intronic	Intron var.	**C**	G	0.7202	0.5305	Protective
rs2961541	*CLEC12A*	12	9964134	Intronic	Intron var.	**C**	T	0.9937	0.3519	Protective
rs193214822	*CLEC12A*	12	9971188	Intronic	Intron var.	**T**	G	0.2497	0.8767	Protective
rs114421141	*CLEC12B*	12	10007247	Intronic	Intron var.	**C**	T	0.8407	0.4977	Protective
rs79967076	*CLEC12B*	12	10004170	Intronic	Intron var.	**A**	G	0.6343	0.6586	Protective
rs112915340	*CLEC12B*	12	10018224	Intronic	Intron var.	**G**	T	0.9492	0.4278	Protective

Our results showed one SNP (rs3774275 in MASP1) significantly associated with pulmonary tuberculosis (*p < 0.05; **p < 0.01). Effect of test allele is also shown.

Joint analysis of phases I and II confirmed our findings, with a highly significant association of rs3774275 with pulmonary tuberculosis (joint analysis OR = 0.61 95%CI = 0.43–086, *p* = 0.0028; see Table 2). The G allele of rs3774275 showed a protective effect (G allele frequency: 39% controls vs. 28% TB patients; Table 3). The GG genotype was twice more frequent in the healthy group (15%) than in the TB group (7%) (see Table 3).

**Table 3:** Distribution of allele and genotype frequencies for SNP rs3774275 (*MASP1*) between controls and tuberculosis patients.

Rs3774275 allele frequencies (*n* = 321)			

Allele	All subjects	Healthy controls	Cases (TB patients)
A	421 (65.6%)	219 (60.8%)	202 (71.6%)
G	221 (34.4%)	141 (39.2%)	80 (28.4%)

**Rs3774275 genotype frequencies (*n* = 321)**			

**Genotype**	**All subjects**	**Healthy controls**	**Cases (TB patients)**

A/A	137 (42.7%)	66 (36.7%)	71 (50.3%)
A/G	147 (45.8%)	87 (48.3%)	60 (42.6%)
G/G	37 (11.5%)	27 (15.0%)	10 (7.1%)

*Shown are the counts and percentages (in brackets) of the 321 samples genotyped for this variant (genotyping of four samples failed)*.

### 3.3 Increased MASP1 Levels in Serum of Tuberculosis Patients

**Figure 1:**
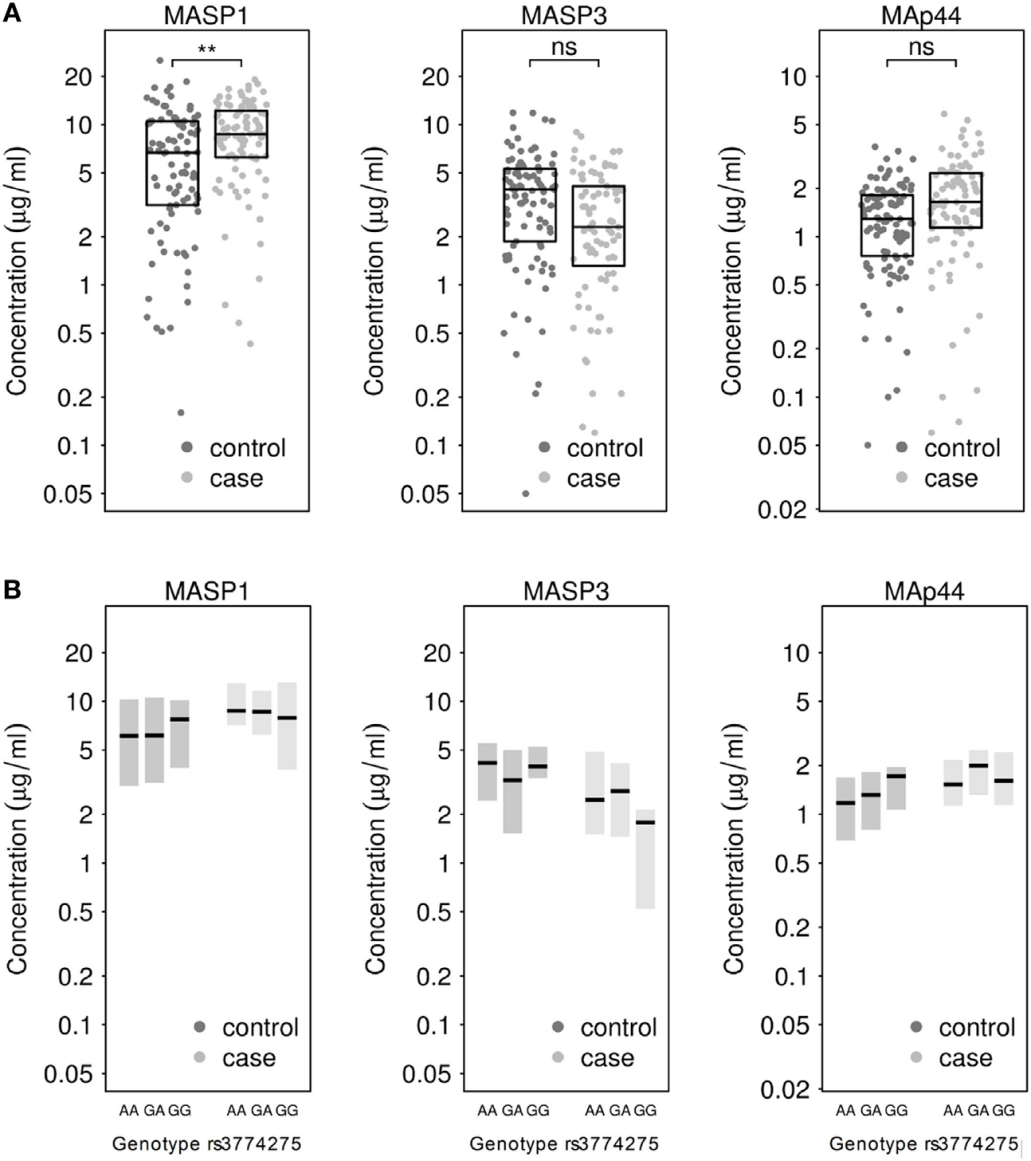
**(A)** Measurement of MASP1, MASP3, and MAp44 levels in serum of healthy controls and tuberculosis patients. Results after adjusting for BMI show significantly higher levels of MASP1 in tuberculosis patients (*T*-test; **p < 0.01) when compared to healthy controls. **(B)** Genotype-dependent distribution of MASP1, MASP3, and MAp44 levels across control and case samples. Shown are median values and 25–75th percentile box plots.

Next, we measured the concentrations of MASP1, MASP3 and MAp44 in the serum. Our results show that the mean concentration of MASP1 was significantly higher in TB patients (median $(\tilde{x})=8.68\;\mu \text{g}/\text{ml}$; mean $(\bar{x})=9.06\;\mu \text{g}/\text{ml}$) than in healthy donors ($\tilde{x}=6.68\;\mu \text{g}/\text{ml}$; $\bar{x}=6.99\;\mu \text{g}/\text{ml}$; see Figure 1A). We could also observe an increase in MAp44 and a decline in MASP3 levels in the serum of cases when compared to controls, although these differences did not reach statistical significance after adjusting for BMI (see Figure 1A). The increase of MASP1 in TB patients might suggest an important role of this protein in the immune response against Mtb.

When the MASP1 serum concentrations were analyzed by genotype in each group, we could observe a slightly higher concentration of MASP1 in healthy donors with a GG-genotype ($\tilde{x}=7.74\;\mu \text{g}/\text{ml}$; $\bar{x}=8.63\;\mu \text{g}/\text{ml}$) as compared to the other 2 genotypes in the same group ($\tilde{x}=6.13\;\mu \text{g}/\text{ml}$; $\bar{x}=6.73\;\mu \text{g}/\text{ml}$; see Figure 1B). Furthermore, the GG-genotype also exhibited higher MAp44 levels in the control group. However, none of the observed genotype-dependent differences reached statistical significance, probably due to the sample size and the proportion of GG-genotype in our population. Nevertheless, the association of rs3774275 with the MASP1 serum concentration has also been documented in other studies (38, 60), where a GG-genotype has been linked with an increased amount of MASP1, ranging between 11 and 13% over the other genotypes. In our population, we observed a similar increase in the GG-genotype of the healthy study group. Median values of MASP1 were 20% higher for the GG-genotype when compared to the other two genotypes (Figure 1B). Interestingly, the MASP1 serum levels of the healthy GG-group were comparable to the concentrations observed in PTB patients.

**Figure 2:**
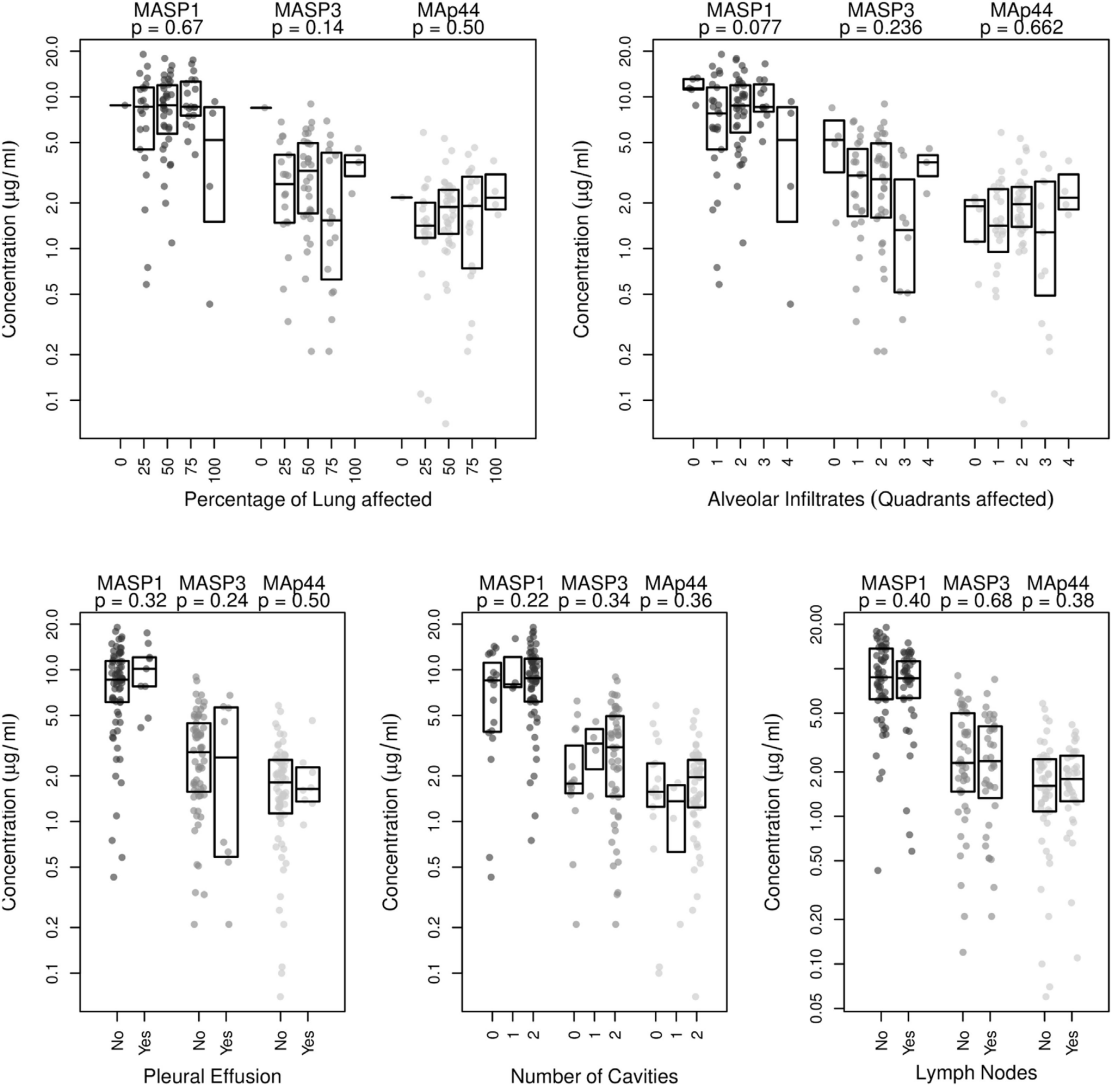
Correlation analysis of MASP1, MASP3, and MAp44 serum concentrations with disease severity, as measured by examination of chest X-rays. Shown are the *p*-values obtained after linear regression analysis for each of the following criteria analyzed: Percentage of lung affected, number of quadrants showing alveolar infiltrates, presence of pleural effusion, number of cavities observed and presence of lymph nodes. Shown are box plots with median values and *p*-values obtained for the correlation with eachMASP1-splicing product.

We next examined radiographic abnormalities in chest X-rays from the TB patients (31) to determine the severity of the disease and to analyze whether the concentrations of MASP1, MASP3, or MAp44 might correlate with the progression of tuberculosis. The radiographic features here analyzed included the number of cavities, the extent of alveolar infiltrates, and the presence of pleural effusion or lymph nodes. These characteristics and the overall percentage of lung affected were previously reported to correlate with TB severity (31). Nevertheless, we could not detect any correlation between the severity-criteria analyzed with neither MASP1 nor MASP3 or MAp44 levels (Figure 2). We also failed to observe a clear correlation of MASPs levels with the BMI of the patients (Figure S3 in Supplementary Material).

### 3.4 MASP1 Levels Influence the Lectin Pathway Complement Activity in vitro

**Figure 3:**
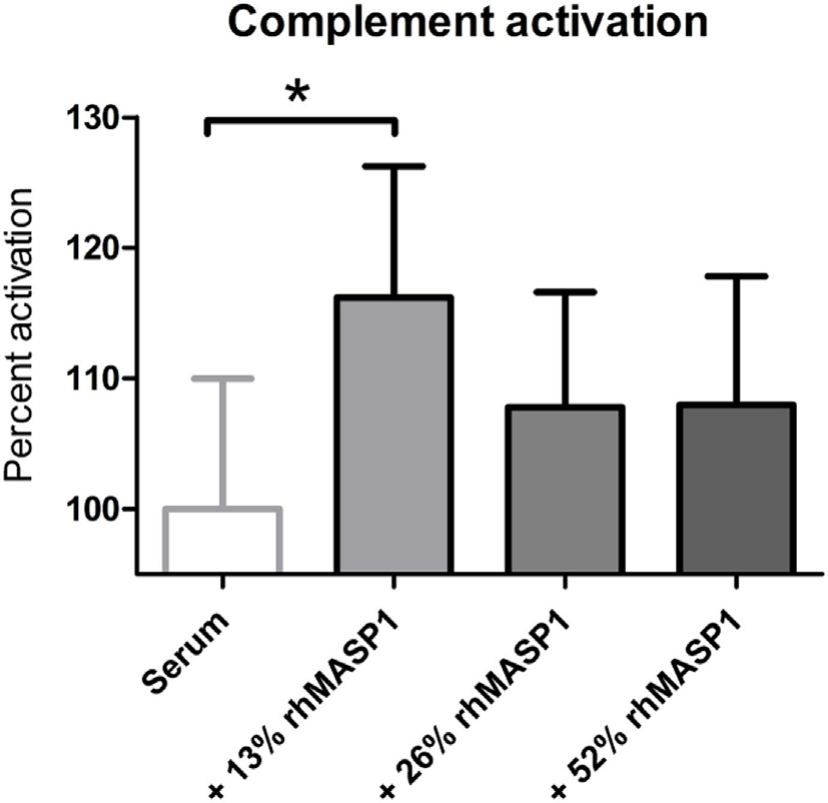
Measurement of lectin pathway complement activation in serum samples (*n* = 6) after addition of rhMASP1. As measured by an MBL pathway complement activation system, an increase in rhMASP1 leads to a slight enhancement of the complement activation in the serum samples (repeated measures ANOVA with Dunnett *post hoc* test; *p < 0.05).

Next, we tested whether small increases in MASP1 concentration, such as those observed in our study, could have any impact on the lectin pathway complement activation. We performed an *in vitro* assay in which we added recombinant human MASP1 to serum samples, and measured the MBL pathway activity using a commercially available ELISA kit. Our results showed a significant increase of the lectin pathway complement activity (*p* < 0.05) after addition of 13% more rhMASP1 to the serum samples (see Figure 3). This suggests that even a small increase in MASP1 concentration is sufficient to improve the efficiency of the MBL-dependent complement activity against pathogens. However, higher concentrations of rhMASP1 did not further increase the complement activation in our *in vitro* system.

## 4 Discussion

In the present work, we used an AmpliSeq-based approach to screen for TB-associated polymorphisms in several genes belonging to the C-type lectin receptor family or their related signaling pathways. Coupling of this NGS approach with a MassARRAY validation phase allowed the identification of a polymorphism in *MASP1* (rs37742752) that was significantly associated with disease susceptibility. Further analysis revealed increased MASP1 levels in serum of tuberculosis patients, constituting the first reported association between tuberculosis and this MBL-associated serine protease.

Previous studies were able to identify tuberculosis-associated variants in a few CTLR genes in different populations, such as for *MRC2, MBL*, or *MASP2* in Chinese populations, *DC-SIGN* variants in African populations, and several variants of *SPA-1, SPA-2*, or *MBL* in diverse populations (29, 30, 61). In our study, we targeted these polymorphisms and expanded the sequencing approach to a total of 33 genes involved in CTLR signaling. We identified a significantly associated polymorphism in the *MASP1* gene, which has not been targeted in previous case-control studies addressing TB susceptibility. MASP1 plays a key role in the activation of lectin pathway of complement (Figure S1 in Supplementary Material). Mtb recognition *via* MBL leads to the activation of associated MASP1 homodimers, which catalyze the activation of MASP2 (17, 62, 63). MASP1 and MASP2 together cleave the C2 and C4 components of the complement and the cleavage products form C3 convertase. MASP1 is responsible for 60% of C2a production needed to generate C3 convertase (63; Figure S1 in Supplementary Material), which further creates a membrane attack complex on bacterial surface ultimately killing the cell, while the by-products of the cascade such as C3b may act as opsonins enhancing the bacterial phagocytosis (22). While the role of C-type lectin MBL polymorphisms in tuberculosis have been studied in several populations (29, 30, 35, 64), not many studies have focused on the other components of the lectin pathway. A recent work found no association between *MBL, Ficolin-1, Ficolin-2*, or *MASP2* variants and TB susceptibility (65). In contrast, Chen et al. could observe a significant impact of *MBL* and *MASP2* polymorphisms upon TB susceptibility in a Chinese population (29, 30). However, the important factor MASP1 has not been included in any studies addressing the genetic predisposition to TB so far. Our study demonstrates for the first time that *MASP1* polymorphism rs3774275 is associated with TB susceptibility.

The SNP rs3774275 is located in the mutually exclusive splicing region in intron 8 of the MASP-1/3 gene and is responsible for the alternative splicing and the regulation of serum levels of MASP1 protein and its splice variants MASP3 and MAp44 (38). Two independent studies by Ammitzboll et al. and Krogh et al. recently showed that rs3774275 is associated with MASP1 serum concentrations (38, 60). In both studies, they observed that the G allele was related to higher MASP1 levels (38, 60). In our study, we also observed higher MASP1 levels in the GG-genotype of the control population, but failed to reach statistical significance, probably due to the small sample size for this minor genotype. However, we were able to detect significantly higher levels of MASP1 in TB patients when compared to healthy controls. As shown by correlation of MASP levels with radiographic evaluations, this increase seems to be independent of the severity of TB.

For long time, MASP2 had been considered the main effector of the lectin pathway of the complement and has been associated with several infectious diseases including Hepatitis C virus (HCV) infection, *Pseudomonas* infection, leprosy, as well as TB (29, 30, 66). Recent investigations on the mechanism of complement lectin-pathway activation suggest that MASP1 plays an even more central role than MASP2 (63). Nevertheless, MASP1 has not yet been studied in association to many infectious diseases. Some studies in HCV infection have demonstrated a high association between MASP1 activity and severe hepatic fibrosis (67, 68). In another study, a synonymous mutation in *MASP1* in the MASP3 serine protease domain was associated with early *Pseudomonas aeruginosa* colonization in cystic fibrosis patients (69). Our study is now the first study to demonstrate an association between MASP1 serum levels and pulmonary TB.

Recent research on MASP1, dissecting its physiological function, has revealed a much broader spectrum of its action than previously assumed. MASP1 is a promiscuous receptor and is shown to bind several ligands. MASP1 can not only activate the complement lectin pathway but also triggers cellular processes such as activation of signaling pathways. Megyeri et al. demonstrated that MASP1 could activate the NFκB, p38-MAPK and Ca^2+^ signaling in endothelial cells *in vitro* by cleaving surface protease activated receptor-4 (PAR-4) (62). Moreover, the p38-MAPK activation in endothelial cells by rMASP1 led to IL-6 and IL-8 secretion along with other cytokines *in vitro* that were able to recruit neutrophils (70). PARs are also expressed on lung epithelium (71), and therefore it may be speculated that high levels of MASP1 may help induce a similar response in lung tissue, activating cellular responses and recruiting phagocytes which may together contribute to bacterial clearance.

The pathognomonic increase of serum MASP1 observed in TB patients in our study reflects the important role that this serine protease plays in the immune response against Mtb. Interestingly, the rs37742752-GG-genotype, which is more frequent in healthy controls, correlates with elevated MASP1 expression as shown in this study and elsewhere (38, 60). Indeed, the MASP1 levels of healthy controls with the GG-genotype ($\bar{x}=8.63\;\mu \text{g}/\text{ml}$) were comparable to those observed in TB patients ($\bar{x}=9.06\;\mu \text{g}/\text{ml}$). It may be hypothesized that intrinsic upregulation of MASP1 (due to genetic predisposition), as observed in healthy controls with the GG-genotype, could play a protective role against infection with Mtb or the development of active TB in latently infected individuals. In the latter case, elevated MASP1 levels might contribute to prevent reactivation of latent Mtb. In this study, we could demonstrate a correlation between MASP1 levels and lectin complement activation *in vitro*. It is likely that, as the amount of MASP1 in the serum increases, more MASP2 is activated and more C2 and C4 molecules are cleaved (see Figure S1 in Supplementary Material), which in turn leads to the formation of more C5b9 complexes and higher opsonization rates. Our results implicate that even a small increase in the amount of MASP1 (+ 13%) can significantly enhance the lectin pathway activity. However, we could not observe any dose dependency when higher concentrations of rhMASP1 were added to the system. The observed saturation might be explained by the interdependency between MASP1 and MASP2 in the activation of the lectin complement system. This activation might reach a plateau when MASP2 becomes the limiting factor, since it is responsible for the cleavage of C4—a process that cannot be engaged by MASP1 (see Figure S1 in Supplementary Material). Further *in vivo* experiments, as well as *in vitro* assays with blood samples of TB patients, are needed to confirm the effect of increased MASP1 levels on the lectin complement pathway and to investigate its potential impact on the phagocytosis and killing of Mtb.

In the present study, we used a customized AmpliSeq approach to screen for relevant polymorphisms in 33 genes. Although this approach led to the identification of a TB-associated polymorphism in *MASP1*, which was strongly supported by the findings in the validation phase, certain limitations of the study have to be acknowledged. One polymorphism that was included in phase II of the study after NGS analysis resulted to be monomorphic during validation. This result might be related to the limitations of the semiconductor sequencing technology. Although the High-Q chemistry of Ion Torrent has largely improved the sequencing output with regard to coverage, noise, and read quality (72), false positives are still possible at loci in proximity of homopolymers. Thus, AmpliSeq approaches should always be coupled with post-validation procedures. An additional challenge of this association study was the potential stratification of the population analyzed. Stratification in the Indian population is expected due to historical ethnic, religious and language barriers existing in the community, which might exert important genetic effects and should be addressed in association studies such as this one (73). Thus, in this study we used the LASER software to correct for potential stratification effects. Ancestry adjustment resulted in a lambda value near 1 and no inflation of the association results (see Figure S2B in Supplementary Material). Finally, although rs3774275 showed a strong association with TB, it is still unclear how this SNP might be linked to other genetic variants or interrelates with other predisposing factors in this multifactorial disease. Moreover, due to the lack of control for asymptomatic Mtb infection in the control group of our study, it cannot be determined whether the rs3774275-GG-genotype confers protection against Mtb infection or resistance against the development of active tuberculosis disease. Considering that over 90% of those who are infected with Mtb remain asymptomatic (74), latently infected individuals should be expected among our control group. Indeed, latent TB prevalence rate in endemic TB countries has been estimated to be as high as 79% (75). Further studies with controls classified for asymptomatic infection will help to clarify the type of protection that is associated with the *MASP1* variant rs3774275.

In summary, in this study we investigated whether genetic variants of CTLR-related genes were associated with TB susceptibility. Our two-stage study allowed the identification of one *MASP1* polymorphism (rs3774275) significantly associated with PTB. MASP1 had been considered the underdog of the lectin-dependent complement activation until recently, when a more prominent role of this protein has been dissected in the lectin pathway (63). Our results, which suggest an important role of *MASP1* variants in tuberculosis, were reinforced by the observation of elevated MASP1 serum levels in PTB patients. The present work contributes to our understanding of host-Mtb interaction and highlights the critical role of the lectin-complement pathway in Mtb pathogenesis.

## Ethics Statement

This study was carried out in accordance with the recommendations of the committee for bio-medical research at the Bhagwan Mahavir Medical Research Centre with written informed consent from all subjects. All subjects gave written informed consent in accordance with the Declaration of Helsinki. The protocol was approved by the institutional ethics committee for bio-medical research at the Bhagwan Mahavir Medical Research Centre, Hyderabad, India (date: March 11, 2011).

## Author Contributions

TK, SG, CF, and HS conceived and designed the study and experiments. TK and SG wrote the manuscript. TK and SG conducted the experiments. TK, SG, MS, DD, LB-A, RS, CF, and HS analyzed the data. SG, AH, RM, GS, and VV recruited the study cohort and collected samples. NA supervised and coordinated the study in India. All authors reviewed and edited the manuscript.

## Conflict of Interest Statement

The authors declare that the research was conducted in the absence of any commercial or financial relationships that could be construed as a potential conflict of interest.
