# Supplementary material for: AmpliSeq Screening of Genes Encoding the C-Type Lectin Receptors and Their Signaling Components Reveals a Common Variant in MASP1 Associated with Pulmonary Tuberculosis in an Indian Population
Source: Front Immunol. 2018 Feb 20;9:242. doi: 10.3389/fimmu.2018.00242 (PMC5826192; doi:10.3389/fimmu.2018.00242)
Supplement: Supplementary file 1 [file Image_1.PDF]

Supplementary Material:

AmpliSeq screening of genes encoding the C-type lectin receptors and their signaling components reveals a common variant in *MASP1* associated with pulmonary tuberculosis in an Indian population

1 SUPPLEMENTARY DATA

2 SUPPLEMENTARY TABLES AND FIGURES

2.1 Figures

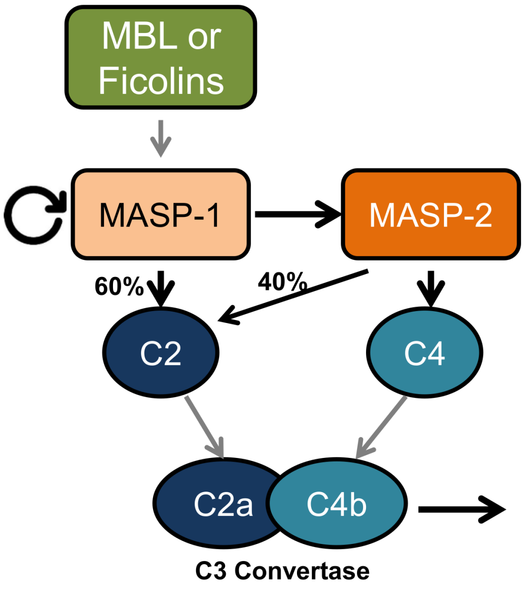

Figure S1: Model of the lectin complement-pathway activation. MASP1 and MASP2 together cleave the C2 and C4 components of the complement and the cleavage products form C3 convertase (adapted from the new model proposed by Heja *et al.*, 2012).

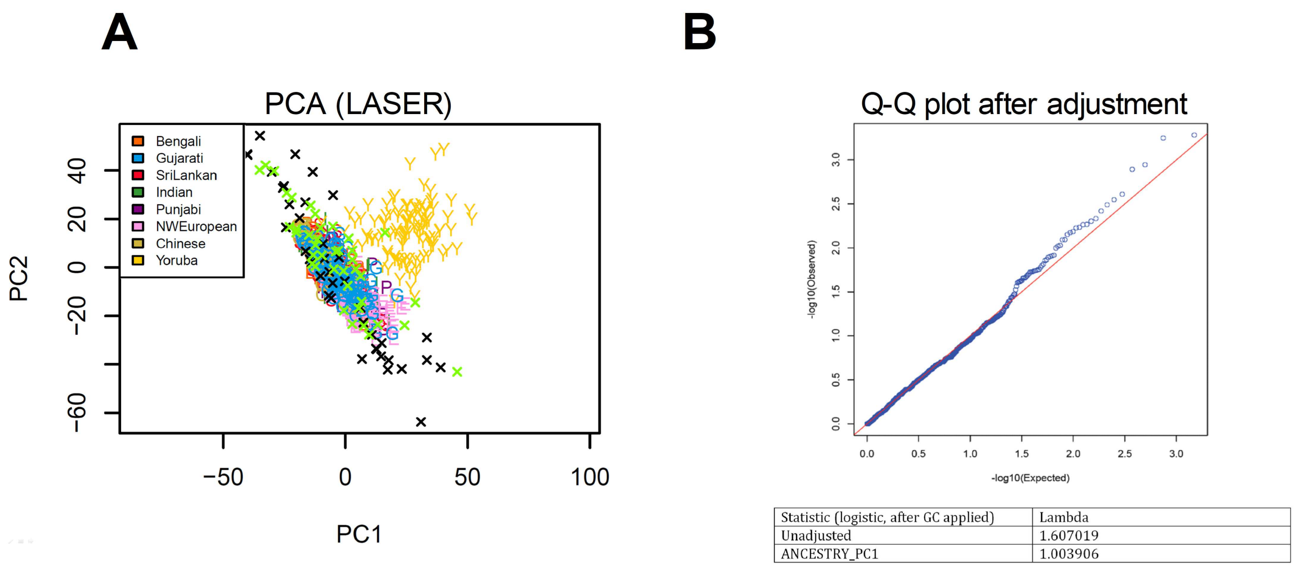

Statistic (logistic, after GC applied)	Lambda
Unadjusted	1.607019
ANCESTRY_PC1	1.003906

Figure S2: **Ancestry adjustment**. **(A)** Initial analysis using `LASER` revealed potential population stratification of our study samples as shown by black (controls) and green (cases) crosses on the PCA space defined by the depicted populations. **(B)** Quantile-quantile plot showing no inflation of association results after stratification adjustment and multiple testing corrections. Shown is also the lambda value before and after adjustment.

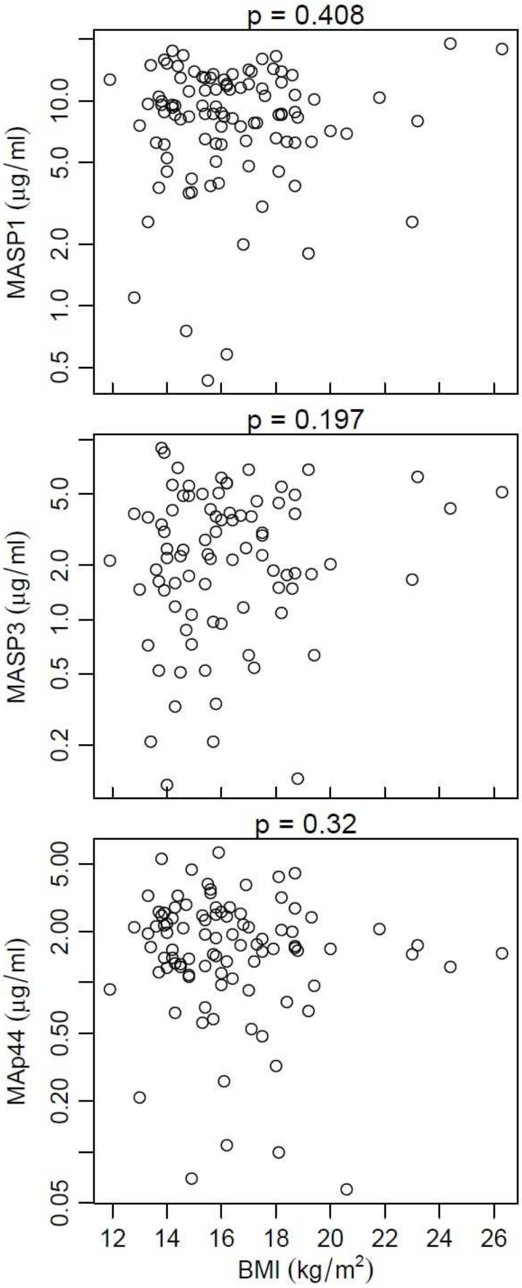

Figure S3: Correlation analysis between MASP1, MASP3 and MAp44 levels and BMI (kg/m^2). Shown are the p-values of the analyses for each splice variant.
