# Supplementary material for: AmpliSeq Screening of Genes Encoding the C-Type Lectin Receptors and Their Signaling Components Reveals a Common Variant in MASP1 Associated with Pulmonary Tuberculosis in an Indian Population
Source: Front Immunol. 2018 Feb 20;9:242. doi: 10.3389/fimmu.2018.00242 (PMC5826192; doi:10.3389/fimmu.2018.00242)
Supplement: Supplementary file 2 [file Table_1.PDF]

Chromosome #	Gene name (SNP rs#)	Start position	End position
chr1	MASP2 rs12711521	11090916	11090917
chr1	MASP2 1630A>G	11106666	11106667
chr1	Bcl10 rs3768235	85733374	85733375
chr1	Bcl10 rs1060843	85742157	85742158
chr1	Bcl10 rs2735593	85742338	85742339
chr1	Bcl10 rs2735591	85744472	85744473
chr2	Langerin rs57302492	71058230	71058231
chr2	Langerin rs13383830	71058306	71058307
chr2	Langerin rs741326	71058834	71058835
chr2	Langerin rs3815556	71059152	71059153
chr2	Langerin rs10204437	71059762	71059763
chr2	Langerin rs17662453	71061107	71061108
chr2	Langerin rs17719042	71061398	71061399
chr2	Langerin rs17006438	71062052	71062053
chr3	PKCdelta rs2230494	53220215	53220216
chr3	MASP1 rs3774275	186965268	186965269
chr3	MASP1 rs190590338	187011884	187011885
chr7	MDL-1 rs1285933	141627148	141627149
chr7	MDL-1 rs1285935	141627938	141627939
chr7	MDL-1 rs2204608	141632351	141632352
chr9	Syk rs290997	93559351	93559352
chr9	Syk rs2991216	93628027	93628028
chr9	CARD9 rs10870077	139263891	139263892
chr9	CARD9 rs121918338	139264814	139264815
chr9	CARD9 rs4077515	139266496	139266497
chr10	MRC-1 rs691005	17962854	17962855
chr10	MRC-1 rs34039386	18138630	18138631
chr10	MBL2 rs10824793	54529479	54529480
chr10	MBL2 rs1800451	54531226	54531227
chr10	MBL2 rs1800450	54531235	54531236
chr10	MBL2 rs5030737	54531242	54531243
chr10	MBL2 rs7095891	54531460	54531461
chr10	MBL2 rs7095891	54531461	54531462
chr10	MBL2 rs11003123	54531533	54531534
chr10	MBL2 rs7096206	54531684	54531685
chr10	MBL2 rs11003125	54532014	54532015
chr10	SP-A2 rs1965708	81317045	81317046
chr10	SP-A2 rs17886395	81318663	81318664
chr10	SP-A2 rs17880349	81318820	81318821
chr10	SP-A2 rs1250943	81318930	81318931
chr10	SP-A2 rs1650232	81319267	81319268
chr10	SP-A1 rs1059047	81371637	81371638
chr10	SP-A1 rs1136450	81371729	81371730
chr10	SP-A1 rs1914663	81371953	81371954
chr10	SP-A1 rs1136451	81372081	81372082
chr10	SP-A1 rs1059058	81373728	81373729
chr10	SP-A1 rs10351	81373770	81373771
chr10	SP-D rs2181204	81704511	81704512
chr10	SP-D rs6413523	81706134	81706135

chr10	SP-D rs721917	81706323	81706324
chr10	SP-D rs721917	81706324	81706325
chr10	SP-D rs726289	81706950	81706951
chr10	SP-D rs2819096	81707612	81707613
chr10	SP-D rs11200982	81732348	81732349
chr10	SP-D rs11200984	81732365	81732366
chr10	SP-D rs11200985	81732652	81732653
chr10	SP-D rs10788338	81733021	81733022
chr10	SP-D rs4255480	81735887	81735888
chr10	SP-D rs3923564	81735980	81735981
chr10	SP-D rs11201000	81736239	81736240
chr12	PTPN6 rs2301262	7055860	7055861
chr12	CLEC4C	7882011	7905201
chr12	CLEC4A	8275228	8291203
chr12	CLEC6A	8607522	8630926
chr12	CLEC4D	8661071	8674962
chr12	CLEC4E	8685901	8694559
chr12	CLEC12A	10102915	10148293
chr12	CLEC1B	10138241	10167023
chr12	CLEC12B	10162226	10171218
chr12	CLEC9A	10182276	10218565
chr12	CLEC1A	10222153	10265226
chr12	CLEC7A	10269376	10283857
chr12	OLR1	10310902	10325737
chr12	PTPN11 rs2301756	112890776	112890777
chr12	PTPN11 rs3741983	112939853	112939854
chr16	CR3 CD11b rs7193943	31271062	31271063
chr16	CR3 CD11b rs1143679	31276811	31276812
chr16	CR3 CD11b rs9929801	31283471	31283472
chr16	CR3 CD11b rs13338129	31284321	31284322
chr16	CR3 CD11b rs9937837	31298938	31298939
chr16	CR3 CD11b rs9938063	31302937	31302938
chr16	CR3 CD11b rs9888879	31310371	31310372
chr16	CR3 CD11b rs8056264	31332654	31332655
chr16	CR3 CD11b rs11150610	31334235	31334236
chr16	CR3 CD11b rs1143683	31336887	31336888
chr16	CD3 CD11b rs4077810	31340908	31340909
chr16	CD3 CD11b rs7193268	31340996	31340997
chr17	MRC2 rs2465412	60708576	60708577
chr17	MRC2 rs8078112	60720361	60720362
chr17	MRC2 rs8068977	60734815	60734816
chr17	MRC2 rs2302242	60742278	60742279
chr17	MRC2 rs7209331	60746273	60746274
chr17	MRC2 rs4968617	60753903	60753904
chr17	MRC2 rs2465429	60766482	60766483
chr17	MRC2 rs2460290	60768845	60768846
chr19	DC-SIGN rs11465413	7805950	7805951
chr19	DC-SIGN rs11465403	7806590	7806591
chr19	DC-SIGN rs1544767	7806867	7806868
chr19	DC-SIGN rs10403018	7807549	7807550

chr19	DC-SIGN rs4804802	7807609	7807610
chr19	DC-SIGN rs8105572	7809326	7809327
chr19	DC-SIGN rs17159889	7809630	7809631
chr19	DC-SIGN rs2287886	7812535	7812536
chr19	DC-SIGN rs2287886	7812536	7812537
chr19	DC-SIGN rs11465366	7812598	7812599
chr19	DC-SIGN rs4804803	7812733	7812734
chr19	DC-SIGN rs11465362	7813141	7813142
chr19	DC-SIGN rs735239	7813267	7813268
chr19	DC-SIGN rs735239	7813268	7813269
chr19	DC-SIGN rs735240	7813335	7813336
chr19	L-SIGN	7830502	7831170
chr19	L-SIGN rs2277998	7831627	7831628
chr19	L-SIGN rs560634	7831952	7831953
chr19	L-SIGN rs874492	7832000	7832001
chr19	L-SIGN rs558705	7832182	7832183
chr19	L-SIGN rs557094	7832285	7832286
chr19	L-SIGN rs3745376	7833689	7833690
chr19	L-SIGN rs1045998	7833993	7833994
chr19	L-SIGN rs15282	7834273	7834274
chr19	DAP10 rs16960862	36390123	36390124
chr21	CR3 CD18 rs684	46306160	46306161
chr21	CR3 CD18 rs2838726	46315090	46315091
chr21	CR3 CD18 rs3788145	46317125	46317126
chr21	CR3 CD18 rs2235133	46321171	46321172
chr21	CR3 CD18 rs2838732	46322944	46322945
chr21	CR3 CD18 rs760459	46328834	46328835
chr21	CR3 CD18 rs3788147	46329668	46329669
chr21	CR3 CD18 rs2838734	46329739	46329740
chr21	CR3 CD18 rs2280965	46330627	46330628
chr21	CR3 CD18 rs2838735	46335281	46335282
chr21	CR3 CD18 rs2838737	46335579	46335580
chr21	CR3 CD18 rs1474552	46337289	46337290
chr21	CR3 CD18 rs9306118	46338402	46338403
chr21	CR3 CD18 rs9976299	46338650	46338651
chr21	CR3 CD18 rs760453	46340511	46340512
chr21	CR3 CD18 rs2070947	46340842	46340843
chr21	CR3 CD18 rs2070946	46341196	46341197
chr21	CR3 CD18 rs2838738	46344425	46344426

Suppl. Table S1:
AmpliSeq Design of the targeted genes and SNPs addressed in this study. Shown are the SNP reference and/or gene names as well as their chromosomal location on the human genome (hg19).
