# Supplementary material for: AmpliSeq Screening of Genes Encoding the C-Type Lectin Receptors and Their Signaling Components Reveals a Common Variant in MASP1 Associated with Pulmonary Tuberculosis in an Indian Population
Source: Front Immunol. 2018 Feb 20;9:242. doi: 10.3389/fimmu.2018.00242 (PMC5826192; doi:10.3389/fimmu.2018.00242)
Supplement: Supplementary file 3 [file Table_2.PDF]

CHR	Chr: position (hg19)	SNP_ID	Allele frequency distribution (nominal p-value)	Association analysis log-add. model (ancestry ADJ - p-value)
10	10:81371953	rs1914663	0.00943	0.07434
12	12:10110431	rs35333643	0.01697	0.1111
12	12:10116631	rs3110948	0.01754	0.0733
3	3:186965268	rs3774275	0.02088	0.06657
12	12:10159846	rs114421141	0.02427	0.1748
12	12:10116733	rs2961541	0.02809	0.1341
12	12:10156769	rs79967076	0.03064	0.1748
12	12:10170823	rs112915340	0.03064	0.1748
12	12:10150905	rs374147676	0.03263	0.09288
12	12:10103208	rs76427726	0.03279	0.1773
12	12:10117091	rs190925857	0.03279	0.1773
12	12:10123787	rs193214822	0.03279	0.1773
12	12:10112586	rs148864420	0.03279	0.1773
12	12:10280862	rs143386125	0.03348	0.06201
2	2:71058835	rs741326	0.03697	0.06597
12	12:10116629	rs3110949	0.04830	0.1341
12	12:10116577	rs648985	0.04830	0.1341
2	2:71058226	rs2080390	0.04965	0.0803
12	12:10158903	rs17205441	0.05621	0.1748
12	12:10170154	rs3736850	0.05621	0.1748
12	12:10160808	rs75972360	0.05643	0.1748
12	12:10156147	rs7132948	0.05655	0.1748
12	12:10151747	rs118077922	0.06186	0.2856
12	12:8615602	rs12099687	0.06186	0.181
12	12:10125497	rs147529241	0.06305	0.1773
12	12:10121476	rs182670340	0.06350	0.1773
12	12:10122346	rs183462894	0.06350	0.1773
12	12:10121072	rs185612837	0.06359	0.1773
12	12:8626362	rs4334073/rs199783308	0.09032	0.1385
12	12:8290569	rs7302963	0.09179	0.1728
12	12:8614238	rs4883148	0.10000	0.1965
12	12:10114753	rs145937548	0.10245	0.1773
19	19:7812536	rs2287886	0.11491	0.2071
12	12:8289877	rs144658267	0.11596	0.0974
2	2:71061108	rs17662453	0.12536	0.1511
12	12:8617976	rs7302011	0.12536	0.07955
12	12:10270773	rs10845048	0.12821	0.152
10	10:54531235	rs1800450	0.13006	0.2152
12	12:10156051	rs116924172	0.13086	0.2396
12	12:7886732	rs77567151	0.13252	0.1654
2	2:71058906	rs3213749	0.14037	0.157
2	2:71058811	rs3213748	0.14037	0.157
2	2:71059153	rs3815556	0.14106	0.157
2	2:71058306	rs13383830	0.14106	0.157
12	12:8626878	rs4883164	0.14140	0.1385
10	10:81706951	rs726289	0.14493	0.2104
12	12:8627924	rs11045619	0.14735	0.1096

12	12:10311916	rs76039690	0.15095	0.1952
10	10:81733022	rs10788338	0.15431	0.3442
12	12:8623095	rs4462413	0.15542	0.1985
12	12:8623101	rs4301857	0.15542	0.1985
12	12:8618971	rs7309596	0.15629	0.1985
12	12:8620339	rs7963053	0.15629	0.1985
12	12:8622599	rs4883157	0.15629	0.1985
12	12:8617757	rs7315590	0.15629	0.1985
12	12:7900184	rs10845821	0.16017	0.2082
12	12:7904111	rs11055602	0.16017	0.2082
10	10:54531685	rs7096206	0.16453	0.2022
10	10:81707613	rs2819096	0.16574	0.386
12	12:10166105	rs57789100	0.16819	0.3009
19	19:7807482	rs4804801	0.17090	0.3682
12	12:8664351	rs7135960	0.17489	0.2016
12	12:8666118	rs11045983	0.18185	0.2289
12	12:10108433	rs676397	0.18185	0.2037
12	12:8662185	rs10841788	0.18300	0.2361
2	2:71061051	rs41285965	0.18545	0.201
19	19:7834274	rs15282	0.18577	0.1528
17	17:60742348	rs2252814	0.18624	0.2436
12	12:8664719	rs28485567	0.19024	0.2398
12	12:8666378	rs11045985	0.19024	0.2398
12	12:7892937	rs11055538	0.19126	0.2235
12	12:8674727	rs7976134	0.19308	0.1966
12	12:10324431	rs35688880	0.19397	0.2927
12	12:10139024	rs112633624	0.19805	0.1951
12	12:10313448	rs11053646	0.20092	0.2266
12	12:10322882	rs2742113	0.20364	0.2537
12	12:10322817	rs2742114	0.20364	0.2537
12	12:10241261	rs117440358	0.20660	0.2361
9	9:93628105	rs2991215	0.20816	0.2884
2	2:71058795	rs3213747	0.20938	0.1785
12	12:8672848	rs138110414	0.20997	0.2259
12	12:10199326	rs373178652	0.20997	0.1912
10	10:81704559	rs2255601	0.22406	0.3129
12	12:7902281	rs73056636	0.22679	0.2428
12	12:8291122	rs1133104	0.22976	0.2526
12	12:8620065	rs7978179	0.23061	0.1985
12	12:8622964	rs116034720	0.23061	0.1985
12	12:8622997	rs114668263	0.23061	0.1985
12	12:8619792	rs4427631	0.23106	0.1985
12	12:8618563	rs7134303	0.23106	0.1985
12	12:8623261	rs4438120	0.23147	0.1985
12	12:8623815	rs4329732	0.23147	0.1985
12	12:8618488	rs4460883	0.23190	0.1985
12	12:10320212	rs3912640	0.23726	0.216
9	9:93628027	rs2991216	0.23782	0.2773
12	12:8622488	rs4883156	0.24056	0.211
12	12:10148203	rs3818343	0.24348	0.3875

12	12:10270813	rs11053593	0.24348	0.279
12	12:10270821	rs11053594	0.24348	0.279
12	12:10270822	rs11053595	0.24348	0.279
12	12:8288403	rs17728942	0.24678	0.2839
12	12:8615809	rs4242889	0.24768	0.2898
21	21:46330674	rs11088969	0.24794	0.3423
12	12:7897184	rs6488614	0.24800	0.3414
12	12:7904154	rs12422412	0.24800	0.3335
12	12:8668281	rs57832572	0.25188	0.2289
12	12:8666801	rs4534636	0.25740	0.2988
16	16:31271063	rs7193943	0.26089	0.329
12	12:10139082	rs574097	0.26137	0.3918
12	12:10275072	rs59913193	0.26431	0.3071
12	12:10315562	rs12827232	0.27007	0.3166
12	12:10133047	rs79246913	0.27253	0.3952
12	12:10245972	rs368830228	0.27337	0.3048
19	19:7833982	rs1045997	0.27337	0.2961
12	12:8284104	rs201561995	0.27648	0.2444
12	12:8609845	rs4242888	0.28078	0.4045
12	12:8611768	rs4264222	0.28078	0.4045
12	12:8612481	rs12300621	0.28078	0.4045
12	12:8612484	rs12302015	0.28078	0.4045
12	12:8612791	rs10770737	0.28078	0.4045
12	12:8613204	rs10770739	0.28078	0.4045
12	12:8610352	rs4402377	0.28078	0.4045
12	12:8610317	rs4623978	0.28078	0.4045
12	12:8610291	rs4255605	0.28078	0.4045
12	12:8607626	rs4882942	0.28078	0.4045
12	12:8609136	rs7968198	0.28078	0.4045
12	12:7882602	rs6488608	0.28457	0.2484
3	3:53220215	rs2230494	0.29896	0.43
9	9:139266405	rs10781499	0.30872	0.3089
9	9:139266496	rs4077515	0.30872	0.3089
2	2:71061399	rs17719042	0.31018	0.3947
2	2:71058184	rs13421115	0.31122	0.2212
12	12:8608482	rs202005669	0.31323	0.3109
12	12:10315695	rs2634159	0.31479	0.3395
12	12:10150961	rs2273987	0.31824	0.4164
12	12:8617982	rs7315873	0.31924	0.1985
12	12:10312291	rs12316150	0.32474	0.3371
12	12:10311166	rs1050289	0.32474	0.3371
12	12:8622107	rs4883155	0.32726	0.1985
12	12:7894192	rs12823478	0.32787	0.4032
12	12:10277113	rs11053613	0.32787	0.2145
12	12:10247561	rs76240769	0.32894	0.3763
12	12:10146004	rs75575903	0.33111	0.4336
12	12:7896655	rs7314869	0.33399	0.3414
12	12:7895628	rs56318901	0.33399	0.3414
10	10:81318663	rs17886395	0.33575	0.3988
12	12:10318348	rs2010655	0.33852	0.416

12	12:10148703	rs659928	0.33974	0.3909
12	12:10325128	rs2742112	0.34223	0.3942
12	12:7897408	rs113955791	0.35000	0.3219
12	12:7903171	rs76947756	0.35000	0.3219
12	12:7904101	rs74903322	0.35000	0.3219
12	12:10143498	rs1359081	0.35307	0.3918
12	12:10320853	rs35335503	0.35713	0.8531
12	12:10319805	rs35492478	0.35758	0.5061
10	10:81704512	rs2181204	0.36247	0.4215
12	12:10315915	rs35311664	0.36388	0.5061
12	12:10273769	rs58924693	0.36570	0.374
12	12:10275529	rs7136680	0.36570	0.374
12	12:10274849	rs56140555	0.36570	0.374
12	12:10274029	rs11053603	0.36570	0.374
12	12:10104003	rs118182007	0.36570	0.3062
12	12:7892802	rs7971286	0.36570	0.4632
12	12:7896564	rs7304977	0.36570	0.4632
12	12:7896605	rs7137970	0.36570	0.4632
12	12:8693789	rs7132177	0.36605	0.4309
12	12:10147722	rs61917158	0.36686	0.3481
12	12:10125814	rs184227507	0.36717	0.3301
12	12:10275769	rs7137840	0.36776	0.2846
12	12:10270938	rs11053597	0.36905	0.279
19	19:7813336	rs735240	0.37615	0.4084
12	12:8608502	rs4628756	0.37773	0.4045
12	12:8610223	rs4638374	0.38301	0.4045
12	12:8611048	rs10743393	0.38301	0.4045
12	12:8610268	rs4435079	0.38301	0.4045
12	12:8607833	rs10734709	0.38361	0.4045
12	12:8611129	rs10770734	0.38415	0.4045
12	12:8610119	rs4641551	0.38415	0.4045
12	12:8611176	rs10770735	0.38415	0.4045
12	12:8611428	rs10743394	0.38415	0.4045
12	12:8611429	rs61920556	0.38415	0.4045
12	12:8613079	rs10770738	0.38415	0.4045
12	12:8609674	rs4497482	0.38415	0.4045
12	12:8609593	rs4883147	0.38415	0.4045
12	12:8608226	rs10770722	0.38472	0.4045
12	12:8611964	rs4623977	0.38765	0.4045
12	12:8612336	rs10770736	0.38765	0.4045
12	12:8613412	rs10770740	0.38765	0.4045
12	12:10154249	rs10772223	0.38981	0.4966
12	12:10122377	rs640817	0.39390	0.4366
12	12:10248927	rs12370211	0.39641	0.7228
12	12:8609795	rs4459385	0.39885	0.5351
12	12:10132335	rs61913543	0.40196	0.4397
12	12:10129599	rs1447875/rs201768158	0.40196	0.4397
12	12:10131044	rs61913542	0.40253	0.4397
21	21:46338403	rs9306118	0.40485	0.48
12	12:10160231	rs7957224	0.41273	0.5215

12	12:10149116	rs4764177	0.41273	0.5195
12	12:10137259	rs684134	0.41273	0.5195
10	10:81319267	rs1650232/rs72659393	0.41432	0.3292
12	12:10320444	rs3741860	0.42211	0.3557
16	16:31340909	rs4077810	0.42698	0.436
12	12:10138708	rs77140517	0.42731	0.5844
12	12:10121927	rs563651	0.42872	0.4614
12	12:10122204	rs560906	0.43142	0.4614
12	12:10124533	rs1797526	0.43142	0.4614
12	12:10147434	rs150432914	0.43925	0.4671
12	12:7882555	rs370237272	0.44261	0.3578
12	12:8291104	rs11043532	0.44261	0.3429
12	12:7897898	rs77511202	0.44462	0.3331
12	12:10226448	rs189753650	0.44479	0.7861
12	12:10109175	rs673173	0.45005	0.8392
12	12:10113083	rs2012504	0.45067	0.4755
12	12:7882038	rs79787224	0.45883	0.4332
12	12:10168563	rs10845019	0.46422	0.4967
12	12:8616149	rs4883154	0.46941	0.2898
12	12:8694267	rs4883167	0.47590	0.6333
12	12:10150614	rs477658	0.47929	0.5066
12	12:10150668	rs1746123	0.47929	0.5066
12	12:10151344	rs7977955	0.47929	0.5066
12	12:10151405	rs7980924	0.47929	0.5066
12	12:10149657	rs582968	0.48209	0.5066
12	12:10150069	rs584856	0.48209	0.5066
12	12:10152679	rs935538	0.48320	0.5066
12	12:10123861	rs525013	0.48496	0.536
12	12:10120548	rs611819	0.48496	0.536
12	12:10120556	rs611821	0.48496	0.536
12	12:10153996	rs12826100	0.48691	0.424
12	12:10123677	rs526157	0.48774	0.536
12	12:10121976	rs562839	0.48774	0.536
12	12:10131865	rs608418	0.48891	0.6497
12	12:10130601	rs570082	0.48891	0.6497
12	12:10127894	rs609216	0.48891	0.6497
12	12:10134345	rs650368	0.49014	0.6396
12	12:7884351	rs17199006	0.49220	0.5193
12	12:10282987	rs11833740	0.49225	0.4645
12	12:7892112	rs117121134	0.49507	0.4183
12	12:10152364	rs11053538	0.49507	0.4164
12	12:10157066	rs12820108	0.49524	0.424
12	12:10148609	rs35890903	0.49524	0.424
12	12:8693689	rs11046135	0.49739	0.6992
12	12:7903567	rs7300199	0.49913	0.4711
12	12:10272593	rs7309123	0.50438	0.5815
1	1:85733374	rs3768235	0.50772	0.4414
12	12:10212093	rs61918595	0.51002	0.9963
12	12:10247896	rs7953120	0.51652	0.471
7	7:141627149	rs1285933	0.51764	0.4173

12	12:10248147	rs7956327	0.51922	0.6867
12	12:10222356	rs7136826	0.52328	0.6009
12	12:10248046	rs7956208	0.52559	0.7117
12	12:10251385	rs3816845	0.52559	0.7117
12	12:10249513	rs10845040	0.52559	0.6116
12	12:10320202	rs11053653	0.52694	0.5115
12	12:10244822	rs7953886	0.52720	0.5957
12	12:10235535	rs10845032	0.52720	0.5957
12	12:10244716	rs7969125	0.52720	0.5957
12	12:10244371	rs6488253	0.52720	0.5957
12	12:10249129	rs11053577	0.52878	0.827
12	12:10249145	rs11053578	0.52878	0.827
12	12:10249204	rs11053579	0.52878	0.827
12	12:10230072	rs1948185	0.52878	0.7117
21	21:46328835	rs760459	0.53142	0.615
12	12:10153815	rs117114115	0.53142	0.4232
12	12:10137296	rs476844	0.53363	0.6066
12	12:10137557	rs479499	0.53363	0.6066
12	12:10129002	rs12298261	0.53363	0.6066
12	12:7888555	rs10505733	0.53368	0.4632
12	12:7890549	rs1894823	0.53368	0.4632
12	12:10116615	rs3110950	0.53408	0.5043
12	12:7890776	rs7311932	0.53408	0.4632
12	12:7894188	rs73056607	0.53408	0.4632
12	12:7896633	rs7305088	0.53408	0.4632
12	12:7898308	rs11055567	0.53408	0.4632
12	12:7901729	rs11055588	0.53408	0.4632
12	12:10271701	rs56371657	0.53408	0.374
12	12:10278489	rs11053617	0.53408	0.374
12	12:7885652	rs7964329	0.53428	0.4632
12	12:7895744	rs7310649	0.53428	0.4632
12	12:7895804	rs7300836	0.53428	0.4632
12	12:7897479	rs116928974	0.53428	0.4632
12	12:7897735	rs6488616	0.53428	0.4632
12	12:7898433	rs11055569	0.53428	0.4632
12	12:10277733	rs11053615	0.53428	0.374
12	12:7898192	rs147781690	0.53497	0.4632
12	12:7894056	rs73056605	0.53698	0.4632
12	12:10136672	rs2961544	0.53874	0.6066
12	12:10130524	rs570931	0.54136	0.6066
12	12:10159793	rs10400564	0.54394	0.5215
12	12:10160081	rs7960084	0.54394	0.5215
12	12:10157932	rs4764187	0.54394	0.5215
12	12:10136199	rs1060648	0.54394	0.5195
12	12:10247571	rs79074764	0.55117	0.4081
7	7:141627939	rs1285935	0.55310	0.5677
10	10:81732366	rs11200984	0.56193	0.5854
10	10:81732349	rs11200982	0.56193	0.5854
10	10:81706973	rs726288	0.56193	0.5854
12	12:8666296	rs73250517	0.56468	0.6855

12	12:10313722	rs11053647	0.56519	0.751
12	12:10129536	rs61913541	0.58013	0.6741
12	12:10155412	rs11053543	0.58504	0.5822
12	12:10150974	rs2273986	0.59127	0.5066
12	12:8693807	rs7306903	0.59325	0.6112
12	12:10112269	rs7957596	0.59325	0.564
12	12:10149406	rs581949	0.59631	0.5066
12	12:10152073	rs4764179	0.59631	0.5066
12	12:10152015	rs4764178	0.59631	0.5066
12	12:10149207	rs59400725	0.59821	0.6056
12	12:10149204	rs35665084	0.59821	0.6056
12	12:10132978	rs623728	0.59864	0.7538
12	12:10126427	rs592206	0.59864	0.7538
12	12:10131034	rs566229	0.60149	0.7538
21	21:46341197	rs2070946	0.60603	0.5937
12	12:10252208	rs6488258	0.60890	0.5974
12	12:10121411	rs478829	0.60951	0.687
12	12:10273166	rs12829123	0.61393	0.6501
12	12:10243143	rs11053575	0.61677	0.6001
12	12:10201646	rs4399401	0.61734	0.7328
12	12:10242865	rs7961436	0.61818	0.6001
12	12:10251445	rs2306894	0.61882	0.6024
10	10:81371698	rs4253512/rs72659390	0.62005	0.3331
12	12:10317246	rs34733039	0.62024	0.5061
12	12:10324081	rs35553961	0.62024	0.5061
10	10:81317064	rs17096771	0.62024	0.4064
12	12:10144863	rs17807046	0.62024	0.3409
12	12:10323471	rs34624528	0.62030	0.5061
12	12:10149947	rs73050636	0.62030	0.503
10	10:81373728	rs1059058	0.62030	0.4064
12	12:8662809	rs7978532	0.62044	0.795
12	12:10251498	rs148373579	0.62062	0.5766
12	12:10231362	rs10845031	0.62195	0.6001
12	12:10138475	rs602320	0.62195	0.4785
12	12:8687704	rs10841846	0.62323	0.5993
12	12:10204478	rs10505750	0.62378	0.7339
12	12:10275336	rs4764271	0.62439	0.7214
17	17:60742279	rs2302242	0.62600	0.6899
12	12:10251157	rs6488256	0.63059	0.7117
12	12:10248289	rs7970083	0.63065	0.8203
12	12:10250366	rs3912645	0.63065	0.7117
12	12:10250729	rs7977902	0.63065	0.7117
12	12:10242161	rs3825300	0.63204	0.952
12	12:10235943	rs3886143	0.63282	0.7205
12	12:10251941	rs11053581	0.63282	0.7117
12	12:10107191	rs4763394	0.63314	0.5071
12	12:8608583	rs4528410	0.63463	0.3252
12	12:10250514	rs376725596	0.63498	0.7117
12	12:10247732	rs4764250	0.63498	0.7117
12	12:10247358	rs4764247	0.63498	0.7117

12	12:10251772	rs2306891	0.63498	0.7117
12	12:10263205	rs2401601	0.63498	0.6563
12	12:10263164	rs2401602	0.63498	0.6563
12	12:10262275	rs2087307	0.63498	0.6563
12	12:10263139	rs2401603	0.63498	0.6563
12	12:10262923	rs2401606	0.63498	0.6563
12	12:10249098	rs10845039	0.63633	0.952
12	12:10233652	rs7980801	0.63633	0.8346
12	12:10241829	rs2277416	0.63633	0.8346
12	12:10263026	rs2401605	0.63696	0.7806
12	12:10263656	rs2401600	0.63696	0.7806
12	12:7889656	rs10845806	0.64048	0.6308
12	12:7896785	rs117012397	0.65333	0.5843
12	12:10273625	rs73068857	0.67337	0.5063
12	12:10184861	rs11835234	0.67947	0.773
12	12:8624495	rs373404974	0.68133	0.5005
12	12:10131684	rs607567	0.68201	0.6066
12	12:10128795	rs2896048	0.68201	0.6066
12	12:10128794	rs2401640	0.68201	0.6066
16	16:31334236	rs11150610	0.68201	0.5809
1	1:85744472	rs2735591	0.68244	0.6796
12	12:10157474	rs4764183	0.68493	0.5869
19	19:7805951	rs11465413	0.68959	0.6734
12	12:10282736	rs11053623	0.69004	0.5132
12	12:10195233	rs1844186	0.69454	0.5364
12	12:10311962	rs10505755	0.70482	0.5917
12	12:10324381	rs11053654	0.70622	0.617
12	12:10316205	rs11053649	0.70855	0.751
12	12:10312691	rs17174597	0.70855	0.751
12	12:10313358	rs3736232	0.70855	0.751
12	12:10313265	rs3736233	0.70855	0.751
12	12:10312289	rs1050283	0.70855	0.751
12	12:10312648	rs13306593	0.70855	0.751
12	12:10311563	rs1050286	0.70855	0.751
12	12:10312914	rs3816844	0.70855	0.751
12	12:10140184	rs636554	0.71002	0.8002
12	12:10279527	rs78646223	0.71129	0.4234
12	12:10315014	rs11053648	0.71133	0.751
12	12:10160527	rs12578560	0.71405	0.69
21	21:46337271	rs56056043	0.71431	0.7531
12	12:10163379	rs1359083	0.71578	0.6962
12	12:10132283	rs7313235	0.71578	0.6741
12	12:10163147	rs1075996	0.71751	0.6962
12	12:10157971	rs10466840	0.71751	0.6962
12	12:10157666	rs4764185	0.71751	0.6962
12	12:10160068	rs7970682	0.71751	0.6962
12	12:10126249	rs7309256	0.71751	0.6741
19	19:7812729	rs79078188	0.71771	0.7364
12	12:10281421	rs17807926	0.71771	0.6692
12	12:10137886	rs1349027	0.71771	0.4778

12	12:10163072	rs1807355	0.71811	0.6962
12	12:10281255	rs16910631	0.71948	0.4645
12	12:10142897	rs679982	0.71990	0.8002
12	12:10139924	rs544783	0.71990	0.6969
12	12:10139021	rs704230	0.71990	0.7684
12	12:10142538	rs678208	0.72161	0.7078
16	16:31298939	rs9937837	0.72161	0.6592
12	12:10183166	rs61918590	0.72359	0.9227
12	12:10149206	rs60114913	0.72400	0.6056
12	12:10124336	rs2984956	0.72471	0.536
12	12:10132878	rs623269	0.72872	0.7538
12	12:10134961	rs519291	0.72872	0.7538
16	16:31336888	rs1143683	0.72942	0.778
12	12:7896427	rs10845818	0.73014	0.6116
12	12:10275684	rs11053608	0.73468	0.7461
12	12:8692843	rs10841856	0.73708	0.9118
12	12:8691142	rs7139227	0.73801	0.5352
12	12:10248964	rs7957278	0.73813	0.7279
12	12:8688075	rs7307228	0.73985	0.712
12	12:10196783	rs6488246	0.74233	0.837
12	12:10196088	rs7297150	0.74233	0.837
12	12:10199083	rs10845028	0.74233	0.837
12	12:10202228	rs1074060	0.74233	0.837
10	10:81319214	rs1059046/rs35576782	0.74241	0.8865
12	12:10277083	rs3901533	0.74328	0.8112
12	12:10276562	rs2078178/rs59180874	0.74328	0.8112
12	12:10169041	rs12824889	0.74399	0.5943
12	12:10122857	rs11053533	0.74542	0.666
12	12:10132773	rs7312943	0.74586	0.666
17	17:60720362	rs8078112	0.74718	0.6951
12	12:10234603	rs12300167	0.74995	0.8346
12	12:10235671	rs10772231	0.74995	0.8346
12	12:10249358	rs11053580	0.74995	0.8346
12	12:10230416	rs7960611	0.74995	0.8346
12	12:10241593	rs1352477	0.74995	0.8346
12	12:10251992	rs10845041	0.74995	0.8346
12	12:10241553	rs5008763	0.74995	0.8346
12	12:10237014	rs6488250	0.74995	0.8346
12	12:10237071	rs3994131	0.74995	0.8346
12	12:10229931	rs1948184	0.75148	0.8346
12	12:10247461	rs4764248	0.75148	0.8346
12	12:10236797	rs4265666	0.75148	0.8346
12	12:10236824	rs3994132	0.75148	0.8346
12	12:10236921	rs6416262	0.75148	0.8346
12	12:10236942	rs6488249	0.75148	0.8346
12	12:10240944	rs2401614	0.75148	0.8346
12	12:10250513	rs374305068	0.75178	0.8243
10	10:81736312	rs77043863	0.76399	0.7938
12	12:10167277	rs637790	0.76548	0.7899
10	10:81318848	rs370004219	0.76548	0.6128

12	12:10165469	rs476474	0.76560	0.7899
10	10:81735981	rs3923564	0.76560	0.5854
10	10:81706135	rs6413523	0.76560	0.5854
10	10:81732653	rs11200985	0.76622	0.5854
12	12:10226463	rs16910035	0.77653	0.9346
12	12:8289226	rs4322490	0.78062	0.9547
12	12:10170727	rs11053548	0.78087	0.8008
12	12:8661951	rs79143728	0.78088	0.609
12	12:8286762	rs7315526/rs77201531	0.78157	0.9547
12	12:8288098	rs10840746	0.79172	0.7642
12	12:8288524	rs10770233	0.79172	0.7642
19	19:7831713	rs367752410	0.80414	0.8523
21	21:46330628	rs2280965	0.80440	0.9104
21	21:46322853	rs2026882	0.81011	0.9054
12	12:10264994	rs1532087	0.81049	0.7201
12	12:10182292	rs71450017	0.81168	0.8009
12	12:7882695	rs17198999	0.81168	0.7622
12	12:7882569	rs17198992	0.81168	0.7622
12	12:7881995	rs3764005	0.81185	0.7622
12	12:10231904	rs16910075	0.82044	0.7021
12	12:7902927	rs117331019	0.82522	0.7127
21	21:46340843	rs2070947	0.83385	0.6127
12	12:8276048	rs4882913	0.83877	0.9059
12	12:10157471	rs4764182	0.84063	0.7984
12	12:8276432	rs7295783	0.84124	0.9059
19	19:7832001	rs874492	0.84384	0.8475
12	12:10109162	rs1447877	0.84547	0.8893
12	12:10158204	rs1868211	0.84603	0.7913
12	12:10162511	rs185772088	0.85061	0.6971
12	12:10312776	rs17174598	0.85282	0.751
12	12:10313134	rs3736234	0.85282	0.751
12	12:10313075	rs3736235	0.85282	0.751
16	16:31302938	rs9938063	0.85311	0.8773
12	12:10157513	rs4764184	0.85325	0.8054
19	19:7831166	rs868875	0.85439	0.7591
19	19:7831628	rs2277998	0.85462	0.7591
12	12:10125493	rs73048876	0.85588	0.7648
12	12:10163375	rs1359082	0.85667	0.8033
12	12:10121089	rs7953702	0.85675	0.9732
12	12:8687897	rs78521210	0.85789	0.617
12	12:10183123	rs61918589	0.85873	0.9227
12	12:10154011	rs643798	0.85988	0.9583
10	10:81317045	rs1965708	0.86147	0.8411
12	12:10145341	rs613871	0.86167	0.9065
12	12:10148031	rs522837	0.86183	0.9117
12	12:10153192	rs564844	0.86229	0.9583
12	12:10156501	rs7134176	0.86249	0.9949
12	12:10146236	rs617956	0.86648	0.7999
12	12:7902657	rs113639661	0.86660	0.491
12	12:8691242	rs4562874	0.86668	0.712

12	12:10250938	rs7313750	0.86673	0.8346
12	12:10165593	rs620449	0.86845	0.6568
12	12:10201778	rs1074059	0.86864	0.837
12	12:10206925	rs7315231	0.86864	0.837
12	12:8687812	rs10841847	0.86864	0.8025
12	12:10207771	rs1488818	0.86946	0.837
12	12:10215582	rs10772230	0.86946	0.837
12	12:10204286	rs10505749	0.86946	0.837
12	12:10131939	rs536947	0.86994	0.6656
12	12:10170161	rs1054611	0.87012	0.7729
12	12:10247829	rs4764251	0.87081	0.7097
12	12:112890776	rs2301756	0.87087	0.8697
12	12:8280908	rs2377422	0.87107	0.8851
12	12:10190537	rs10845025	0.87277	0.9326
12	12:10186284	rs6488244	0.87439	0.9326
12	12:10247117	rs10845034	0.87447	0.9498
12	12:10247137	rs10845035	0.87447	0.9498
12	12:10151179	rs7980702	1.00000	0.3514
21	21:46337226	rs56332357	1.00000	0.58
21	21:46317126	rs3788145	1.00000	0.6616
12	12:10195202	rs4237956	1.00000	0.6625
12	12:10129694	rs686148	1.00000	0.6656
21	21:46322945	rs2838732	1.00000	0.6891
12	12:10158376	rs1868212	1.00000	0.6962
12	12:10231393	rs11053573	1.00000	0.7021
12	12:10224336	rs11838264	1.00000	0.7042
12	12:10265095	rs1001449	1.00000	0.7042
12	12:10229281	rs11053572	1.00000	0.7042
12	12:10124373	rs2961542	1.00000	0.7116
12	12:10202239	rs111554735	1.00000	0.7554
12	12:8283536	rs4424738	1.00000	0.7642
12	12:8287961	rs10840744	1.00000	0.7642
12	12:8288026	rs12099836	1.00000	0.7642
12	12:10189624	rs190497665	1.00000	0.7666
12	12:10236586	rs3887490	1.00000	0.7673
12	12:10271055	rs7959451	1.00000	0.7691
21	21:46322938	rs186089759	1.00000	0.7693
12	12:10109648	rs7302029	1.00000	0.772
19	19:7831953	rs560634	1.00000	0.7723
12	12:10191719	rs5011267	1.00000	0.773
12	12:10186622	rs4764208	1.00000	0.7749
12	12:7902283	rs142503346	1.00000	0.7879
12	12:10158924	rs4764188	1.00000	0.7896
16	16:31276811	rs1143679	1.00000	0.7944
12	12:10209711	rs10505751	1.00000	0.7979
12	12:10202639	rs11053569	1.00000	0.7979
12	12:10275533	rs4764272	1.00000	0.799
12	12:10127344	rs553104	1.00000	0.8112
12	12:10139025	rs113815287	1.00000	0.8184
12	12:10281847	rs7311598	1.00000	0.819

12	12:8612928	rs61922366	1.00000	0.8235
12	12:10246296	rs7979762	1.00000	0.8343
12	12:10191746	rs10845026	1.00000	0.8355
19	19:7809327	rs8105572	1.00000	0.8576
12	12:10273804	rs79522375	1.00000	0.8588
12	12:8283348	rs10840731	1.00000	0.8594
10	10:54531534	rs11003123	1.00000	0.8594
12	12:10241554	rs61918622	1.00000	0.8717
12	12:10187941	rs10845023	1.00000	0.8761
16	16:31310372	rs9888879	1.00000	0.8773
12	12:10144246	rs531425	1.00000	0.8783
12	12:10271087	rs16910526	1.00000	0.8811
12	12:10156825	rs7305223	1.00000	0.8884
12	12:10109449	rs1447879	1.00000	0.8893
12	12:10109086	rs1447876	1.00000	0.8893
12	12:10108854	rs11525545	1.00000	0.8893
12	12:10104289	rs7957464	1.00000	0.8893
12	12:10255442	rs77549003	1.00000	0.8914
12	12:10113258	rs999185	1.00000	0.8919
12	12:10114476	rs1535652	1.00000	0.8919
12	12:10117369	rs12230244	1.00000	0.8919
12	12:10114475	rs1535651	1.00000	0.8919
12	12:10111199	rs7314437	1.00000	0.8919
12	12:10113662	rs6488238	1.00000	0.8919
12	12:10110742	rs7306520	1.00000	0.8919
12	12:10110724	rs7303131	1.00000	0.8919
12	12:10201254	rs76883345	1.00000	0.8985
12	12:10204371	rs1488817	1.00000	0.8985
12	12:10192809	rs78064987	1.00000	0.8985
12	12:10203926	rs1488816	1.00000	0.8985
12	12:10199357	rs10505748	1.00000	0.8985
12	12:10199853	rs17807434	1.00000	0.8985
12	12:10156646	rs7305054	1.00000	0.899
12	12:10202685	rs59462804	1.00000	0.9007
12	12:10191482	rs5011269	1.00000	0.9151
12	12:10191483	rs5011268	1.00000	0.9151
12	12:10160241	rs510630	1.00000	0.9158
12	12:10163583	rs590429	1.00000	0.9158
12	12:10126098	rs80042268	1.00000	0.9161
12	12:10280845	rs11053620	1.00000	0.9183
12	12:10155706	rs1447888	1.00000	0.9184
12	12:10191459	rs5011270	1.00000	0.9262
21	21:46338651	rs9976299	1.00000	0.9266
12	12:10280852	rs11053621	1.00000	0.9273
12	12:8284102	rs77283400	1.00000	0.9289
12	12:8622267	rs145656638	1.00000	0.9311
21	21:46335282	rs2838735	1.00000	0.934
12	12:10279526	rs79314785	1.00000	0.9344
12	12:10253808	rs60567086	1.00000	0.9346
12	12:10224552	rs56000846	1.00000	0.9346

12	12:10246314	rs6488254	1.00000	0.9372
12	12:10246431	rs7962341	1.00000	0.9372
12	12:10246484	rs7976945	1.00000	0.9372
12	12:10246675	rs5012088	1.00000	0.9372
12	12:10246711	rs952546	1.00000	0.9372
12	12:10126141	rs770750	1.00000	0.9388
12	12:10191494	rs2054888	1.00000	0.9392
12	12:10136297	rs1323461	1.00000	0.9416
12	12:10248608	rs374624895	1.00000	0.9483
19	19:36390123	rs16960862	1.00000	0.9502
12	12:10124659	rs201144260	1.00000	0.9531
12	12:8279784	rs4883072	1.00000	0.9531
12	12:10215654	rs11831360	1.00000	0.9546
12	12:10199257	rs10505747	1.00000	0.9546
12	12:10210736	rs61459404	1.00000	0.9546
12	12:10213234	rs73259794	1.00000	0.9546
12	12:8284656	rs11043488	1.00000	0.9547
12	12:8287048	rs11043498	1.00000	0.9547
12	12:8277556	rs11043470	1.00000	0.9593
10	10:54532014	rs11003125	1.00000	0.9602
12	12:7885689	rs7964345	1.00000	0.9617
7	7:141627899	rs13222726	1.00000	0.9621
10	10:81373674	rs4253526/rs148138544	1.00000	0.9625
21	21:46344426	rs2838738	1.00000	0.9662
12	12:10184298	rs113211113	1.00000	0.9663
12	12:10183263	rs11053561	1.00000	0.9663
21	21:46328779	rs367847454	1.00000	0.9679
12	12:10190953	rs10772227	1.00000	0.9714
12	12:10189652	rs4474534	1.00000	0.9714
10	10:54531461	rs7095891	1.00000	0.9775
10	10:54531226	rs1800451	1.00000	0.9866
10	10:81372081	rs1136451	1.00000	0.9872
12	12:10247544	rs3912642	1.00000	0.9914
12	12:10186409	rs6488245	1.00000	0.9922
19	19:7813268	rs735239	1.00000	0.9947
12	12:10156852	rs7134681	1.00000	0.9949

Suppl. Table S2:
List of known SNPs that passed the filters applied for sequencing depth (>20 reads/sample), Minor Allele Frequency (MAF>0.02) and Hardy-Weinberg Equilibrium. Shown are the SNP_ID, chromosomal position (hg19) and the p-values obtained after association analysis.
