# Supplementary material for: AmpliSeq Screening of Genes Encoding the C-Type Lectin Receptors and Their Signaling Components Reveals a Common Variant in MASP1 Associated with Pulmonary Tuberculosis in an Indian Population
Source: Front Immunol. 2018 Feb 20;9:242. doi: 10.3389/fimmu.2018.00242 (PMC5826192; doi:10.3389/fimmu.2018.00242)
Supplement: Supplementary file 4 [file Table_3.PDF]

SNP_ID	Location				Type of mutation	Alleles			Log. regression (p-values)	
	Gene	Chr	Position (GRCh38)	Gene location		Maj	Min	Allele freq (p-nominal)	UNADJ.	ADJUSTED + GC
**rs741326**	CD207	2	70831704	Exonic	Missense	G	A	0.03697	**0.02093**	**0.06597**
**rs2080390**	CD207	2	70831095	Exonic	Synonymous	C	T	0.04965	**0.02805**	**0.0803**
**rs3774275**	MASP1	3	187247480	Intronic	Intron variant	A	G	0.02088	**0.02122**	**0.06657**
**rs1914663**	SFTPA1	10	79612197	Intronic	Intron variant	C	T	0.00943	**0.02501**	**0.07434**
**rs143386125**	CLEC7A	12	10128263	Intronic	Intron variant	C	A	0.03348	**0.01908**	**0.06201**
**rs76427726**	CLEC12A	12	9950609	Intronic	Intron variant	T	C	0.03279	**0.09018**	**0.1773**
**rs35333643**	CLEC12A	12	9957832	Intronic	Intron variant	A	G	0.01697	**0.04537**	**0.1111**
**rs148864420**	CLEC12A	12	9959987	Intronic	Intron variant	C	A	0.03279	**0.09018**	**0.1773**
**rs648985**	CLEC12A	12	9963978	Intronic	Intron variant	G	C	0.04830	**0.05992**	**0.1341**
**rs3110949**	CLEC12A	12	9964030	Intronic	Intron variant	T	C	0.04830	**0.05992**	**0.1341**
**rs3110948**	CLEC12A	12	9964032	Intronic	Intron variant	A	C	0.01754	**0.02449**	**0.0733**
**rs2961541**	CLEC12A	12	9964134	Intronic	Intron variant	T	C	0.02809	**0.05992**	**0.1341**
**rs190925857**	CLEC12A	12	9964492	Intronic	Intron variant	T	C	0.03279	**0.09018**	**0.1773**
**rs193214822**	CLEC12A	12	9971188	Intronic	Intron variant	G	T	0.03279	**0.09018**	**0.1773**
**rs114421141**	CLEC12B	12	10007247	Intronic	Intron variant	T	C	0.02427	**0.08831**	**0.1748**
**rs79967076**	CLEC12B	12	10004170	Intronic	Intron variant	G	A	0.03064	**0.08831**	**0.1748**
**rs112915340**	CLEC12B	12	10018224	Intronic	Intron variant	T	G	0.03064	**0.08831**	**0.1748**
**rs374147676**	CLEC1B	12	9998306	Exonic	Missense	C	A/T	0.03263	**0.03482**	**0.09288**

Suppl. Table S3. Detailed list of the 18 candidate SNPs included in phase II after association analysis of the AmpliSeq data. P-values are shown for the differences observed in allele frequency and genotype distribution between TB patients and healthy controls (nominal and ancestry adjusted).
